# Structural-functional connectivity decoupling reveals neural differences associated with subtypes of nonsuicidal self-injury among female depressive adolescents

**DOI:** 10.3389/fpsyt.2026.1775531

**Published:** 2026-02-19

**Authors:** Lan Hu, Kena Li, Hui He, Shaoqing Li, Nan Qiu, Guocheng Zhao, Tingyu Hu, Yong Chen, Cheng Luo

**Affiliations:** 1 The Clinical Hospital of Chengdu Brain Science Institute, MOE Key Lab for Neuroinformation, University of Electronic Science and Technology of China, Chengdu, China; 2 High-Field Magnetic Resonance Brain Imaging Key Laboratory of Sichuan Province, School of Life Science and Technology, University of Electronic Science and Technology of China, Chengdu, China

**Keywords:** adolescents, functional magnetic brain imaging (fMRI), neuroimaging, nonsuicidal self-injury (NSSI), structural-functional connectivity coupling

## Abstract

**Background:**

Nonsuicidal self-injury (NSSI) is a complex behavior prevalent among adolescents, particularly females and those with depression. The DSM-5 introduced recommended diagnostic criteria for NSSI, yet many adolescents engaging in NSSI do not meet these standards. The neurobiological distinctions between adolescents with NSSI who fulfill the DSM-5 criteria (NSSI+) and those who do not (NSSI-) remain unclear.

**Methods:**

Sixty-three female depressive adolescents (40 NSSI+, 23 NSSI-) and 35 healthy controls (HCs) were included and underwent resting-state functional magnetic resonance imaging, diffusion tensor imaging and high-resolution T1-weighted imaging. We explored differences in brain structure-function interactions by applying structural-functional connectivity (SC-FC) coupling analysis using multimodal neuroimaging data. Partial Spearman’s correlation analyses were used to identify association between SC-FC coupling and clinical features.

**Results:**

The NSSI+ group had notably distinct SC-FC coupling in task-positive network regions including decreased SC-FC coupling (greater decoupling) in the left dorsolateral superior frontal gyrus and increased coupling in the bilateral medial precuneus and right opercular inferior frontal gyrus, as compared to the NSSI- group. Moreover, the NSSI+ group displayed widespread coupling abnormalities across multiple networks compared to the HC group, while the NSSI- group only differed in the left dorsolateral superior frontal gyrus. Correlational analyses linked decoupling indices to several clinical features, particularly in the right subgenual anterior cingulate among the NSSI- participants.

**Conclusions:**

These findings indicate that SC-FC coupling patterns distinguish NSSI subtypes in depressed female adolescents, with more severe NSSI associated with altered coupling in prefrontal, precuneus, and inferior frontal regions involved in executive control and attentional processing. The right subgenual anterior cingulate cortex—showing multiple clinical correlations—emerges as a potential target for early intervention of NSSI behavior. These findings highlight the utility of SC-FC coupling as a neural marker for NSSI subtyping and intervention planning.

## 1 Introduction

Nonsuicidal self-injury (NSSI; all abbreviations are listed in **Table 1**), defined as the deliberate infliction of harm to one’s body tissue without suicidal intent (1), predominantly manifests in adolescents and often co-occurs with depression, with the most common form being cutting (2–4). The global lifetime prevalence of NSSI among adolescents was estimated to be around 20% (5, 6), peaking in the age range of 15–17 years (7). Previous research indicates that females were more likely to engage in NSSI behaviors than males among adolescents in both community- (2, 8) and clinical- population (4). The emergence and persistence of NSSI during adolescence exert various adverse effects on individuals’ physical and mental health, including may heighten the risk for emotional dysregulation and even increased suicidal tendencies (9–11), thereby increasing the burden on social services and health resources.

**Table 1:** Abbreviation.

Abbreviation	Full name
NSSI	nonsuicidal self-injury
FC	functional connectivity
DSM-5	the fifth version of the Diagnostic and Statistical Manual of Mental Disorders
SC-FC	structural-functional connectivity
MRI	magnetic resonance imaging
NSSI+	suprathreshold nonsuicidal self-injury
NSSI-	subthreshold nonsuicidal self-injury
HC	healthy controls
CDI	Children’s Depression Inventory
SCAS-S	Spence Children’s Anxiety Scale - Short Version
BIS-11	Barratt Impulsiveness Scale
ERQ	Emotion Regulation Questionnaire
TAS-20	Toronto Alexithymia Scale - 20 items
OSI	Ottawa Self-injury Inventory
BSS	Beck Scale for Suicide Ideation
fMRI	functional magnetic resonance imaging
EPI	echo-planar imaging
TR	repetition time
TE	echo time
FA	flip angle
3D FSPGR	three-dimensional fast spoiled gradient-echo
DTI	diffusion tensor imaging
DPABI	Data Processing Assistant for Brain Imaging
FWHM	full-width at half-maximum
BET	Brain Extraction Tool
BNA	Brain Network Atlas
SDI	structural-decoupling index
ANOVA	analysis of variance
LSD	least significant difference
FDR	false discovery ratio
sgACC	subgenual anterior cingulate cortex
STG	left superior temporal gyrus
SFG	superior frontal gyrus
IFG	inferior frontal gyrus
DAN	dorsal attention network
VAN	ventral attention network
FPN	frontoparietal network
DLPFC	dorsolateral prefrontal cortex
SMA	supplementary motor area
DMN	default mode network
rTMS	repetitive transcranial magnetic stimulation

Neuroimaging studies have significantly advanced our understanding of the neural underpinnings of NSSI in adolescents. Accumulating evidence suggests that NSSI is not merely a behavioral symptom, but a condition marked by measurable disruptions in brain connectivity—encompassing both functional networks and structural pathways. At the functional level, previous research has established that adolescents with NSSI exhibit widespread functional connectivity (FC) abnormalities, particularly in cortical-subcortical communication pathways. For example, some studies have identified aberrant resting-state FC between the amygdala and prefrontal areas and dorsal anterior cingulate cortex (12), as well as between the orbitofrontal cortex and insula (13), the latter predicting future self-injury frequency in depressed youth. Such findings extend to reward-related circuits, where dysregulated connectivity may underlie the addictive nature of some NSSI behaviors (14). Task-based studies further reveal deficits in reward processing and negative emotion regulation among adolescents with NSSI, linked to altered connectivity involving regions such as the orbitofrontal cortex (15) and the amygdala (12). Notably, FC patterns may be linked to the clinical prognosis of NSSI. For example, prefrontal-amygdala connectivity has been shown to predict responsiveness to both psychological and pharmacological interventions, suggesting its potential as a neural marker for forecasting treatment outcomes in NSSI (16, 17). On a broader scale, network-level disruptions are also evident. Adolescents with NSSI have been found to exhibit reduced coherence within major intrinsic networks such as the default mode and salience networks (18), suggesting widespread integration deficits in systems supporting self-referential and attentional processing. Structurally, adolescents with NSSI demonstrated widespread white matter microstructural deficits, with longer duration of self-injury associated with more severe impairment in white matter integrity (19). Together, neuroimaging evidence consistently points to extensive disruptions in brain connectivity among adolescents with NSSI, which may be closely linked to impairments in emotional processing, cognitive control, and related functions.

To foster further research into NSSI, the fifth edition of the Diagnostic and Statistical Manual of Mental Disorders (DSM-5) has incorporated NSSI into its recommended diagnoses, recognizing it as a distinct clinical disorder separate from other self-destructive behaviors. Among the proposed criteria, self-injurious behavior should occur on 5 or more days in the past year. The DSM-5 also outlines functional and intentional aspects relevant to diagnosis. However, both community-based and clinical studies indicate that many adolescents who self-injure do not meet the criteria for NSSI outlined in the DSM-5. Meta-analyzes suggest that among community-based adolescent populations, those who have engaged in self-injurious behaviors more than five times in their lifetime account for less than 30%, and only about 5% have engaged in such behaviors more than 10 times (2). Similarly, data from a public hospital in Mexico indicate that approximately 40% of adolescent patients presenting with self-injury do not satisfy the diagnostic criteria for NSSI (20).

Existing research has begun to differentiate between occasional and repetitive self-injury, which roughly align with subthreshold and suprathreshold frequencies relative to DSM-5 recommendations. For example, Brunner et al. (21) found that social factors were associated with occasional but not repetitive self-harm. Liu et al. (22) reported that impulsivity moderated the relationship between emotion dysregulation and NSSI only in the repetitive NSSI group. Additionally, Liu et al. (23) observed that both maladaptive cognitive schemas and emotion dysregulation mediated the link between stressful life events and addictive features of NSSI in adolescents with repetitive NSSI—a pattern not seen in those with occasional NSSI. Biologically, a recent study introduced the concept of “subthreshold NSSI” to describe individuals engaging in self-injury fewer than five times per year. This work revealed abnormalities in low−frequency oscillations in the left thalamus and posterior cingulate cortex, altered FC within the left thalamus, and reduced abundance of Prevotellaceae bacteria in the gut among subthreshold NSSI patients (24). While this study represents the first exploration of biological alterations in individuals who do not meet the DSM−5 diagnostic threshold for NSSI, it did not directly compare subthreshold and suprathreshold NSSI groups. Consequently, whether and how adolescents who meet the DSM-5 diagnostic threshold for NSSI differ biologically from those who do not still remains unclear.

As previously noted, NSSI is increasingly understood as a disorder characterized by disruptions in brain connectivity. However, the majority of earlier neuroimaging studies have examined brain alterations in NSSI from either a structural or a functional perspective in isolation. Researchers have established that structural and functional connectivity are highly interdependent—brain activity both emerges from and is constrained by underlying structural pathways (25, 26). Notably, a seminal finding revealed that the human brain exhibits a gradual structural-functional gradient, shifting from strong coupling in highly myelinated sensorimotor cortices toward greater decoupling in less myelinated associative regions (27). This organizational principle aligns with gradients observed in other biological modalities, such as gene expression (28, 29) and microstructural properties (30). These insights stem from structural-functional connectivity (SC-FC) coupling analysis based on magnetic resonance imaging (MRI). Conceptually, SC-FC coupling reflects the degree to which functional signaling depends on anatomical structure, often quantified as the smoothness of functional signals over the structural graph. Preti et al. (27) further demonstrated that brain regions supporting primary sensory and motor functions exhibit stronger SC-FC coupling, whereas areas involved in higher-order cognitive and affective processes—such as memory, reward, and emotion—show greater decoupling.

Importantly, altered SC-FC coupling has been implicated in the neuropathology of several psychiatric disorders, including bipolar disorder (31) and adolescent depression (32). Notably, Xu et al. (32) identified distinct SC-FC coupling profiles in limbic regions between depressed adolescents with and without NSSI behavior. Thus, SC-FC coupling analysis offers a novel integrative framework for investigating the neural substrates of NSSI, shifting the focus from isolated connectivity measures to the dynamic interplay between brain structure and function.

Therefore, from a structural-functional integration perspective, this study aims to investigate—through SC-FC coupling analysis—whether adolescents whose NSSI meets the DSM−5 diagnostic threshold exhibit distinct neurobiological substrates compared to those with subthreshold NSSI. Given the known heterogeneity of NSSI related to gender and comorbid psychiatric conditions, this work focuses specifically on female adolescents with depression. By leveraging multimodal neuroimaging, the study seeks to provide new insights into the neurobiological distinctions between clinically defined subtypes of NSSI in youth.

## 2 Materials and methods

This experiment conformed to the requirements of the Declaration of Helsinki and was approved by the Ethics Committee of the Chengdu Fourth People’s Hospital. All participants and their guardians had thoroughly read and signed the informed consent form. All participants were recruited from December, 2021, to September, 2024.

### 2.1 Participants

Female adolescents with depression who had engaged in NSSI within the past year by self-report were recruited from the Chengdu Fourth People’s Hospital. NSSI was defined as deliberate injury to one’s body tissue without suicidal intent and for purposes not socially sanctioned (33). Inclusion criteria were: (1) age 10–18 years; (2) right-handedness and completion of at least primary education; (3) at least one episode of NSSI in the past year; and (4) a diagnosis of major depressive disorder according to DSM-5 criteria or clinically assessed depressive state, with no additional DSM-5 psychiatric diagnoses present. All participants underwent diagnostic assessment by an experienced, trained psychiatrist and were further classified into a suprathreshold NSSI (NSSI+) group or a subthreshold NSSI (NSSI-) group based on DSM-5 suggested diagnostic criteria for NSSI. Additionally, female healthy controls (HC) with no current or lifetime DSM-5 psychiatric disorders and no history of self-injurious behavior were recruited through advertisements. Exclusion criteria for all participants were (1) the presence or history of severe medical or neurological disorders, (2) substance use disorder, brain trauma, or loss of consciousness, (3) intellectual impairment; and (4) any contraindication to MRI scanning.

### 2.2 Psychological assessments

All participants were evaluated for their sociodemographic and clinical characteristics. Depressive and anxiety symptoms were assessed utilizing the Chinese version of the Children’s Depression Inventory (CDI) (34) and the Spence Children’s Anxiety Scale - Short Version (SCAS-S) (35), respectively. Given prior evidence linking NSSI behaviors to impulsivity, emotional dysregulation, and alexithymia, we assessed these traits for all participants using the Barratt Impulsiveness Scale (BIS-11) (36), the Emotion Regulation Questionnaire (ERQ) (37), and the Toronto Alexithymia Scale - 20 items (TAS-20) (38). The ERQ is consisted of the reappraisal subscale and the expressive suppression subscale.

Furthermore, NSSI behavioral characteristics were assessed using the Ottawa Self−Injury Inventory (OSI) (39). This is a self-report inventory used to measure the occurrence, frequency, functions, and addictive features of NSSI behavior. Specifically, the four functions factors (internal emotion regulation, social influence, external emotion regulation, and sensation-seeking) were applied to measure the functions of NSSI behavior at both baseline and within the past month, and the addictive factor was utilized to evaluate the addictive features of NSSI behavior. Additionally, the Beck Scale for Suicide Ideation (BSS) was utilized to assess the intensity of suicidal ideations for each NSSI participant in the recent week as well as during their most depressive period (40).

### 2.3 MRI acquisition and pre-processing

All neuroimaging data were acquired using a 3.0T Siemens scanner operated by certified MRI technicians. Resting-state functional magnetic resonance imaging (fMRI) data acquisition was performed under eye-closed condition, with participants instructed to maintain head stillness while remaining awake. Functional images were obtained using a standard echo-planar imaging (EPI) sequence with the following parameters: 34 axial slices; repetition time (TR) = 2000 ms; echo time (TE) = 30 ms; flip angle (FA) = 90°; matrix size = 64 × 64; slice thickness = 4.4 mm. Each participant completed 255 whole-brain volume acquisitions, resulting in a total scan duration of 510 seconds. High-resolution T1-weighted structural images were acquired using a three-dimensional fast spoiled gradient-echo (3D FSPGR) sequence with the following parameters: 152 sagittal slices; TR = 2,300 ms; TE = 2.32 ms; matrix size = 256 × 256; isotropic voxel size = 0.9 mm³. Diffusion tensor imaging (DTI) data were obtained using a diffusion-weighted spin-echo EPI sequence with 64 diffusion encoding directions. Acquisition parameters included: TR = 8,500 ms; TE = 76 ms; FA = 90°; matrix size = 128 × 128; slice thickness = 2 mm; diffusion direction = 64; b = 1,000 s/mm^2^.

Functional image preprocessing was implemented through a Data Processing Assistant for Brain Imaging (DPABI) toolbox (http://rfmri.org/dpabi), comprising the following sequential steps: (1) DICOM-to-NIfTI format conversion; (2) removal of the first five time points to allow for signal equilibration and participant adaptation to the scanning noise; (3) temporal slice timing correction and spatial realignment for head motion correction; (4) spatial normalization to MNI152 standard template space; (5) spatial smoothing with a 6 mm full-width at half-maximum (FWHM) Gaussian kernel; (6) nuisance signal regression incorporating 24-parameter head motion profiles, cerebrospinal fluid signals, and white matter signals; (7) temporal band-pass filtering (0.01-0.08 Hz).

Structural T1-weighted images were preprocessed using FreeSurfer’s automated recon-all pipeline (https://surfer.nmr.mgh.harvard.edu/fswiki/recon-all). DTI preprocessing was performed using FSL tools (https://fsl.fmrib.ox.ac.uk/fsl/fslwiki/FSL), including: (1) non-brain tissue removal via a Brain Extraction Tool (BET); (2) eddy current and motion artifact correction using FSL’s eddy tool; (3) tensor estimation for fractional anisotropy and mean diffusivity computation; (4) probabilistic diffusion parameter estimation through Markov Chain Monte Carlo sampling in FSL’s Bayesian framework. Following MRI preprocessing, participants exhibiting excessive head motion (translation ≥3 mm or rotation ≥3°) were excluded. All structural MRI scans were reviewed by a board-certified radiologist, confirming the absence of clinically significant brain abnormalities across all participants.

### 2.4 SC-FC coupling analysis based on graph Fourier transform

In this study, we utilized the Brainnetome Atlas (BNA) template (41) to extract regional time series. The BNA is a connectome-based brain atlas that integrates white matter fiber tractography with large-scale FC profiles, offering detailed structural and functional connectivity patterns as well as task-related functional annotations. As such, this template aligns well with the objective of our study—to investigate disease mechanisms from an integrated structural-functional connectivity perspective. We extracted time series for 246 brain regions based on this template from the preprocessed resting-state fMRI data for each participant, constructing a 246 × T matrix. Additionally, we estimated the number of white matter fiber tracts between each pair of brain regions for each participant using probabilistic tractography based on DTI data, resulting in a 246 × 246 matrix. Subsequently, we calculated the gray matter volume for these 246 brain regions based on T1-weighted images and normalized the previously obtained matrix by dividing each column by the corresponding gray matter volume, yielding a 246 × 246 structural adjacency matrix A.

For the adjacency matrix A, we first performed symmetric normalization and subtracted the normalized adjacency matrix from the standard identity matrix I to obtain the standard Laplacian operator *L*, as shown in Equation (1): $L=I-D^{-1/2}AD^{-1/2}$, where D is the diagonal matrix of A with elements equal to the row sums of A. We then performed eigendecomposition on the Laplacian operator *L*, with the eigenvalues *Λ* interpreted as different frequencies and the eigenvectors *U* as structural connectivity harmonics, serving as frequency components, as shown in Equation (2):  LU = ΛU. Concurrently, we treated the functional signal time series for the 246 brain regions as graph signals and used the obtained eigenvectors as the basis for Graph Fourier Transform to convert the functional signals *S_t_* from the spatial domain to the spectral domain and vice versa, as shown in Equation (3): $\hat{S_{t}}=U^{T}S_{t}\;,\;S_{t}=U\hat{S_{t}}$.

Then, we performed graph signal filtering on the functional signals, decomposing them into high-coupling and decoupling components. We defined the cutoff frequency as the frequency that divides the average energy spectral density into two equal parts. Using the N × N matrix $U^{\left(low\right)}$ (containing the first C columns of eigenvectors from U and the remaining N - C columns of zero vectors) to filter out high-frequency signals, while $U^{\left(h\text{i}gh\right)}$ (containing the first C columns of zero vectors and the remaining N - C eigenvectors from U) was used to filter out low-frequency signals. Therefore, the filtered high-frequency and low-frequency signals were obtained through Equations (4) and (5), respectively: $S_{t}^{C}=U^{\left(low\right)}U^{T}S_{t}$ and $S_{t}^{D}=U^{\left(h\text{i}gh\right)}U^{T}S_{t}$.

Ultimately, we quantified the coupling relationship between structure and function for each brain region using the ratio of the L2 norm of the filtered low-frequency signal *S^D^* to the L2 norm of the filtered low-frequency signal *S^C^*, termed the structural-decoupling index (SDI), as shown in Equation (6): $SDI=\log_{2}\frac{∥s_{t}^{D}∥}{∥s_{t}^{C}∥}$. Positive SDI values indicate SC-FC decoupling, while negative SDI values indicate SC-FC coupling.

**Figure 1:**
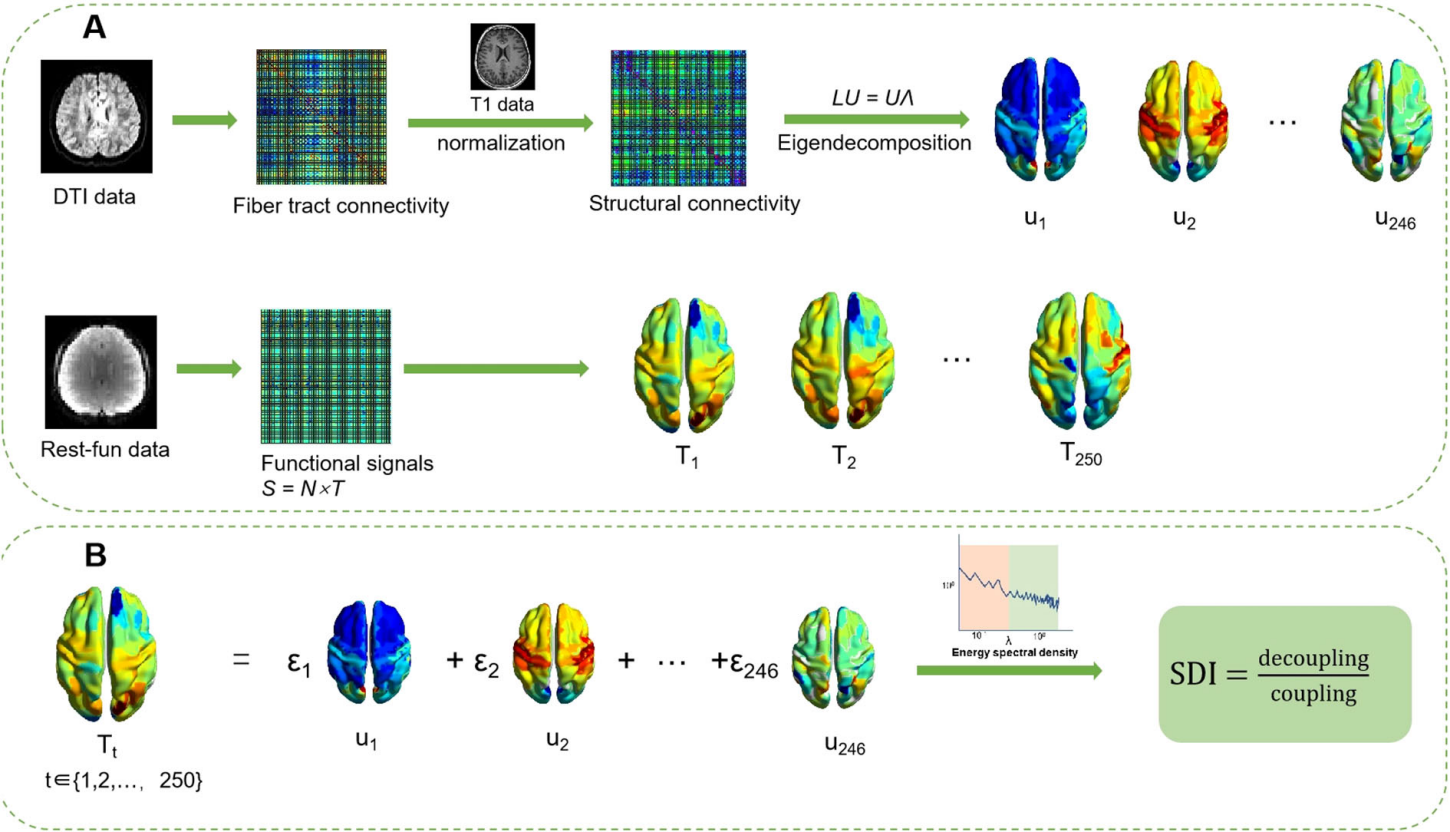
Method pipeline. **(A)** Eigendecomposition of the structural connectivity from DTI and T1 data; resting-state functional signals are represented as an N × T matrix. **(B)** Coupling mechanism: Functional signals are modeled as a linear combination of structural eigenmodes. Based on the energy spectral density, they are partitioned into coupled and decoupled components, with the SDI defined as SDI $=\frac{\text{decoupling}}{\text{coupling}}$. DTI, diffusion tensor imaging; SDI, structural-decoupling index.

The SC-FC coupling analysis in the current study followed the methodological pipeline described by Preti and Van De Ville (27) and detailed processing was provided in **Figure 1**.

### 2.5 Statistical and correlational analyzes

The variables on the demographic and clinical characteristics were compared among the NSSI+, NSSI-, and HC groups using one-way analysis of variance (ANOVA), and *post-hoc* least significant difference (LSD) tests were used to identify the between-group differences. In the comparisons of the subscales of the OSI and the BSS between the two NSSI groups, two-sample *t* tests were employed to identify differences. Statistical significance was accepted at a threshold of *p* < 0.05. All data were analyzed using the Statistical Package for the Social Sciences 25.0 (SPSS Version 25.0; IBM Corporation, Armonk, NY, USA).

For the neuroimaging data, one-sample *t* tests were firstly conducted on the SDI values of NSSI+, NSSI-, and HC groups, respectively. Subsequently, a non-parametric permutational one-way ANOVA with age and in-scanner head motion as covariates was performed on the SDI values of these three groups to identify inter-group differences among them. Randomization was repeated 10,000 times and significance was set at *p* < 0.01 with false discovery ratio (FDR) correction. For the brain regions with significant inter-group differences, *post-hoc t* tests using 10,000 times permutations with age and in-scanner head motion as covariates were employed to measure significant between-group differences, and a significant level was set at *p* < 0.01. Finally, to investigate whether there were associations between the SDI values in brain regions with significant inter-group differences and the clinical variables, the partial Spearman’s correlation analyzes using age and in-scanner head motion as covariates were employed for both the NSSI+ and NSSI- groups, respectively, and significance was set at *p* < 0.05 and FDR correction was applied for multiple comparisons.

## 3 Results

### 3.1 Demographic and clinical characteristics

Forty NSSI+ patients, 23 NSSI- patients, and 35 HCs were finally included in the study. No significant inter-group differences were observed in age and educational level among the three groups (*p*s > 0.05). Detailed demographic and clinical information is provided in **Table 2**.

**Table 2:** The demographic and clinical data of the NSSI+, NSSI-, and HC groups.

Demographic/Clinical variables		NSSI+ (40)	NSSI- (23)	HC (35)	F/T	*P v*alue
Demographic characteristics						
Age (*mean years ± SD*)		14.87 ± 1.54	14.82 ± 1.67	14.82 ± 2.19	0.008	0.992^a^
Educational level (*mean years ± SD*)		9.20 ± 1.57	8.87 ± 1.60	8.83 ± 2.18	0.455	0.636^a^
Current diagnosis -n (%)						
Major depressive disorder		36 (90%)	20 (87%)	/		
Depressive state		4 (10%)	3 (13%)	/		
Medications						
Current medication ***- n(%)***		20 (50%)	8 (35%)	/		
Antidepressants		15 (38%)	4 (17%)	/		
Mood stabilizer		2 (5%)	1 (4%)	/		
Antipsychotics		10 (25%)	2 (9%)	/		
Benzodiazepines		3 (8%)	1 (4%)	/		
unknown		6 (15%)	2 (9%)	/		
Clinical measurements						
CDI		33.65 ± 6.91	31.91 ± 6.63	9.77 ± 5.90	144.413	<0.001 a^,***^
SCAS-S		54.43 ± 8.87	49.83 ± 11.04	33.54 ± 8.66	49.229	<0.001 a^,***^
BIS-11		63.51 ± 13.69	56.42 ± 16.78	36.30 ± 12.64	36.040	<0.001 a^,***^
ERQ_reappraisal subscale		19.58 ± 6.62	22.78 ± 6.62	30.94 ± 5.67	31.406	<0.001 a^,***^
ERQ_expressive suppression subscale		16.13 ± 4.74	18.13 ± 4.73	14.29 ± 5.27	4.259	0.017 ^a,*^
TAS-20		59.78 ± 6.10	54.26 ± 9.55	38.14 ± 11.43	54.758	<0.001 a^,***^
BSS						
The recent week		16.95 ± 8.91	13.96 ± 7.01	/	1.382	0.172^b^
The most depressive period		27.98 ± 7.09	23.43 ± 6.97	/	2.461	0.017^b,*^
*NSSI*						
OSI_function						
Baseline						
	Total score	46.15 ± 16.86	37.35 ± 18.09	/	1.943	0.057 ^b^
	Internal emotion regulation	19.60 ± 7.26	15.26 ± 7.69	/	2.235	0.029 ^b,*^
	External emotion regulation	11.85 ± 6.83	10.61 ± 7.75	/	0.661	0.511 ^b^
	Social influence	7.28 ± 3.05	6.04 ± 3.32	/	1.494	0.140 ^b^
	Sensation seeking	5.08 ± 3.63	3.17 ± 3.24	/	2.080	0.042 ^b,*^
	No reason	2.25 ± 1.46	2.09 ± 1.59	/	0.412	0.682 ^b^
Within the past month						
	Total score	45.90 ± 20.35	34.26 ± 19.73	/	2.210	0.031 ^b,*^
	Internal emotion regulation	18.85 ± 8.23	13.57 ± 8.08	/	2.470	0.016 ^b,*^
	External emotion regulation	11.05 ± 7.13	9.91 ± 6.98	/	0.614	0.541^b^
	Social influence	7.05 ± 3.48	5.74 ± 3.54	/	1.430	0.158 ^b^
	Sensation seeking	4.78 ± 4.04	2.43 ± 2.87	/	2.444	0.017 ^b,*^
	No reason	2.18 ± 1.57	1.65 ± 1.61	/	1.262	0.212 ^b^
OSI_ addictive properties		14.83 ± 5.91	9.00 ± 6.31	/	3.675	0.001^b,**^

NSSI+, suprathreshold nonsuicidal self-injury; NSSI-, subthreshold nonsuicidal self-injury; HC, healthy controls; SD, standard deviation; CDI, Children’s Depression Inventory; SCAS-S, Spence Children’s Anxiety Scale-Short Version; BIS-11, Barrett Impulsiveness Scale; ERQ, Emotion Regulation Questionnaire; TAS-20, Toronto Alexithymia Scale-20 items; BSS, Beck Scale for Suicide Ideation; OSI, Ottawa Self-injury Inventory. ^a^Statistic computed using one-way analysis of variance; ^b^Statistic computed using two-sample *t* test; *p < 0.05, **p < 0.01, ***p < 0.001.

There were significant inter-group differences on the scores of the CDI, SCAS-S, BIS-11, ERQ_reappraisal subscale, ERQ_expressive suppression subscale, and TAS-20 among the three groups (*p*s < 0.017) (see **Table 2** for details). In *post-hoc* analyzes, when compared to the HC group, the NSSI+ and NSSI- groups scored significantly higher on the CDI score, the SCAS-S score, the BIS score, and the TAS-20 score (*p*s < 0.001), while scored significantly lower on the ERQ_reappraisal subscale (*p* < 0.001), and the NSSI- group scored significantly higher on the ERQ_expressive suppression subscale (*p* = 0.005) while no significantly difference was observed on this subscale score between the NSSI+ group and the HC group (*p* = 0.111); when compared to the NSSI- group, the NSSI+ group scored significantly higher in the TAS-20 scale (*p* = 0.023), while there were no significant differences on other clinical variables between the NSSI+ and NSSI- groups (*p* > 0.05) (see **Table 3** for details in *post-hoc* analyzes).

**Table 3:** The results in LSD *post-hoc* two-sample *t* test analyzes on clinical data between groups.

Clinical scale	Comparison	Mean difference	*t*	*p*
CDI				
	NSSI+ *vs*. HC	23.879	15.873	<0.001^***^
	NSSI- *vs*. HC	22.142	12.692	<0.001^***^
	NSSI+ *vs*. NSSI-	1.737	1.021	0.310
SCAS-S				
	NSSI+ *vs*. HC	20.882	9.650	<0.001^***^
	NSSI- *vs*. HC	16.283	6.489	<0.001^***^
	NSSI+ *vs*. NSSI-	4.599	1.880	0.063
BIS-11				
	NSSI+ *vs*. HC	27.210	8.325	<0.001^***^
	NSSI- *vs*. HC	20.115	5.307	<0.001^***^
	NSSI+ *vs*. NSSI-	7.095	1.920	0.058
ERQ_reappraisal subscale				
	NSSI+ *vs*. HC	-11.368	-7.801	<0.001^***^
	NSSI- *vs*. HC	-8.160	-4.829	<0.001^***^
	NSSI+ *vs*. NSSI-	-3.208	-1.947	0.054
ERQ_expressive suppression subscale				
	NSSI+ *vs*. HC	1.840	1.610	0.111
	NSSI- *vs*. HC	3.845	2.902	0.005^**^
	NSSI+ *vs*. NSSI-	-2.005	-1.553	0.124
TAS-20				
	NSSI+ *vs*. HC	21.632	10.250	<0.001^***^
	NSSI- *vs*. HC	16.118	6.586	<0.001^***^
	NSSI+ *vs*. NSSI-	5.514	2.311	0.023^*^

LSD, Least Significant Difference; NSSI+, suprathreshold nonsuicidal self-injury; NSSI-, subthreshold nonsuicidal self-injury; HC, healthy controls; CDI, Children’s Depression Inventory; SCAS-S, Spence Children’s Anxiety Scale-Short Version; BIS-11, Barrett Impulsiveness Scale; ERQ, Emotion Regulation Questionnaire; TAS-20, Toronto Alexithymia Scale-20 items. *p < 0.05, **p < 0.01, ***p < 0.001.

The BSS was used to evaluate the intensity of suicidal ideation. During the most depressive period, the intensity of suicidal ideation in the NSSI+ group was significantly higher than that in the NSSI- group (*p* = 0.017). There was no statistically significant difference in the current suicidal ideation between the two NSSI groups (*p* = 0.172). For detailed information, refer to **Table 2**.

With respect to the functions of NSSI as measured by the OSI, whether it was for the baseline or within the past month of NSSI behavior, the NSSI+ group scored significantly higher on the factors of internal emotion regulation and sensation seeking than the NSSI- group (*p*s < 0.042). Furthermore, the NSSI+ group had a significantly higher total score on the functions of NSSI behavior within the past month compared to the NSSI- group (*p* = 0.031), while no significant differences were found between the two groups on other factors of NSSI functions (*p* > 0.05). Additionally, the NSSI+ group scored significantly higher on the addictive features of NSSI behavior than the NSSI- group (*p* = 0.001). See **Table 2** for details.

### 3.2 SC-FC coupling analyzes

**Figure 2:**
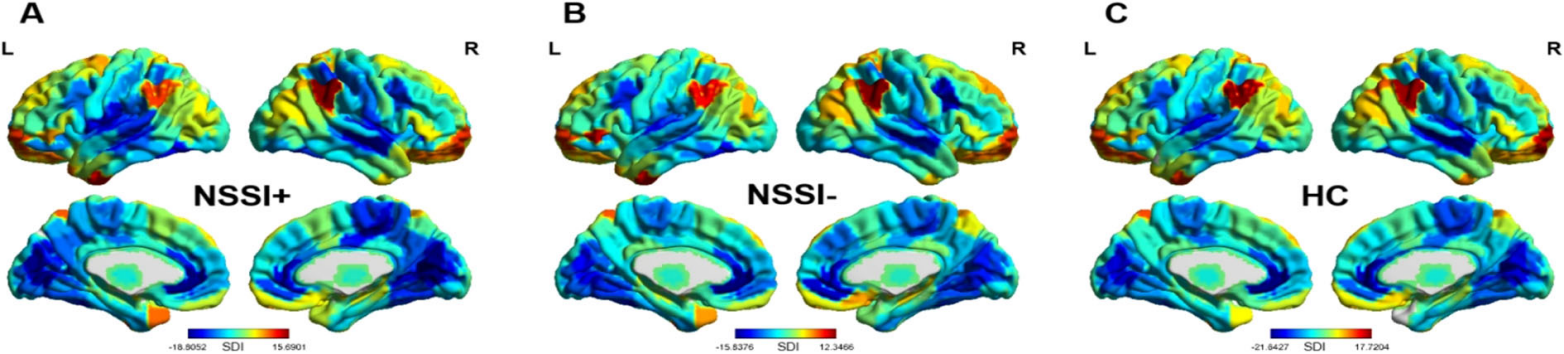
One-sample *t* tests of the SDI values among each group (**A** for the NSSI+ group, **B** for the NSSI- group and **C** for the HC group). The results of the one-sample *t* tests revealed the spatial organization of the coupling strength between the structure and function among each group. Positive SDI values indicate structural-functional decoupling, while negative SDI values indicate structural-functional coupling. Warm colors represent higher decoupling, and cool colors represent higher coupling. The color bar indicates *t* value based on one-sample *t* test. L, left; R, right; NSSI+, suprathreshold nonsuicidal self-injury; NSSI-, subthreshold nonsuicidal self-injury; HC, healthy controls; SDI, structural-decoupling index.

The patterns of SC-FC coupling across the brain in each group as revealed by one-sample *t* tests of the SDI values were similar and consistent with the reports by Preti and Van De Ville (27). The lower-level sensory regions, such as the visual, auditory, and somatosensory areas, were found to be more coupled between function and structure with negative SDI values, while higher-level cognitive regions, such as the frontal orbital, parietal, and temporal areas, were more decoupled with positive SDI values (see **Figure 2**).

**Figure 3:**
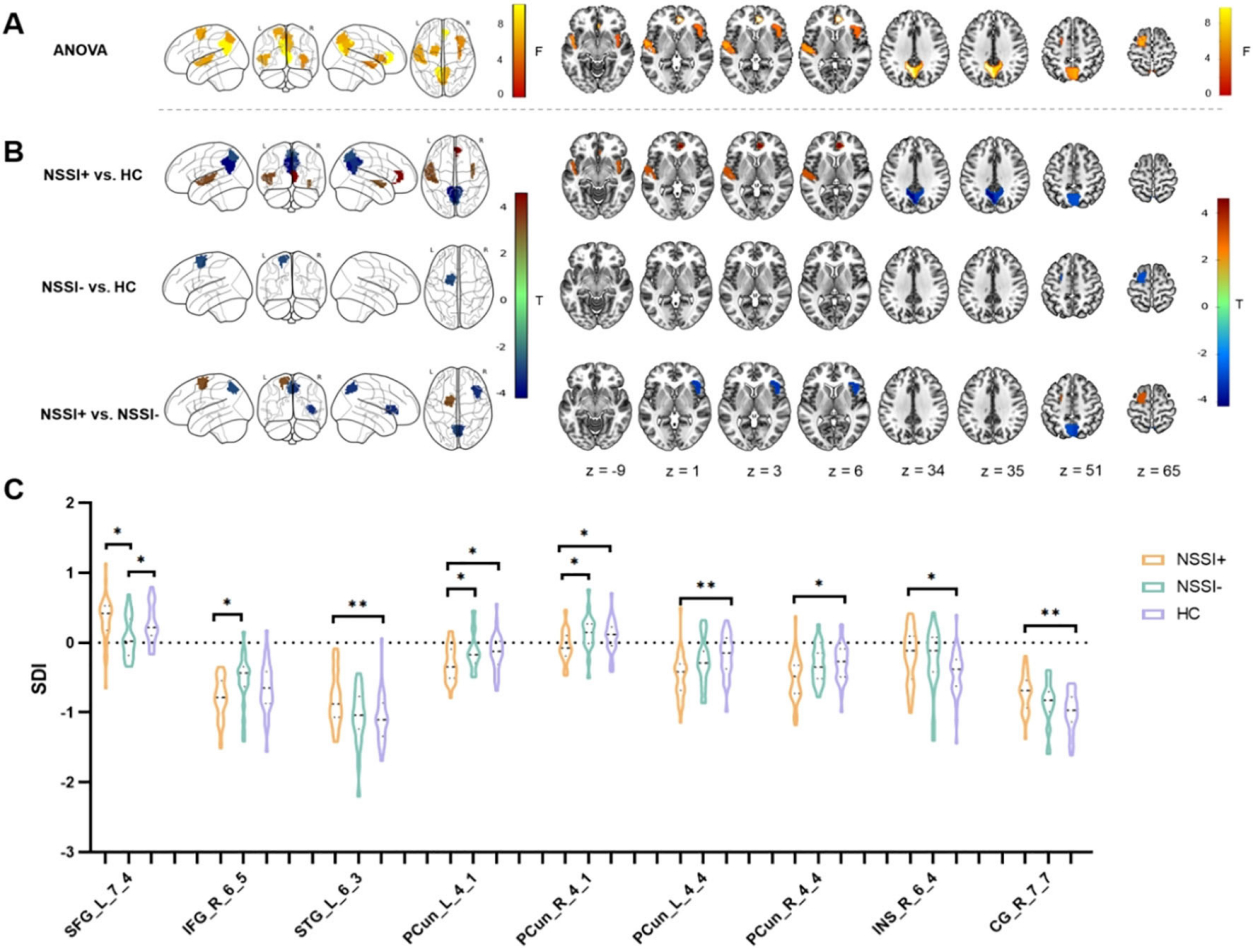
Group effects of the SDI values among the three groups and *post-hoc* comparisons. **(A)** Significant inter-group differences were identified in the right sgACC, bilateral precuneus subregions, left dorsolateral SFG, left STG, right IFG, and right insula (*p* < 0.01, 10,000 permutations, FDR corrected). **(B)***Post-hoc* comparisons of the brain regions with significant inter-group differences (*p* < 0.01, 10,000 permutations). As for the color bar of T values, warm colors represent decreased coupling and cool colors represent increased coupling. **(C)** The violin plots showed detailed comparisons of *post-hoc* analyzes (*p* < 0.01, 10,000 permutations). Specifically, as compared to the HC group, the NSSI+ group showed significantly increased SC-FC coupling in bilateral precuneus subregions (labeled as the PCun_L_4_1, PCun_R_4_1, PCun_L_4_4, PCun_R_4_4), along with decreased SC-FC coupling in the right sgACC (labeled as the CG_R_7_7), left STG (labeled as the STG_L_6_3), and right insula (labeled as the INS_R_6_4), and the NSSI- group showed increased coupling in the left dorsolateral SFG (labeled as the SFG_L_7_4); as compared to the NSSI- group, the NSSI+ group showed decreased SC-FC coupling in the left dorsolateral SFG (labeled as the SFG_L_7_4), and increased coupling in the bilateral medial precuneus (labeled as the PCun_L_4_1, PCun_R_4_1) and the right opercular IFG (labeled as the IFG_R_6_5). ANOVA, analysis of variance; L, left; R, right; NSSI+, suprathreshold nonsuicidal self-injury; NSSI-, subthreshold nonsuicidal self-injury; HC, healthy controls; SDI, structural-decoupling index; sgACC, subgenual anterior cingulate cortex; SFG, superior frontal gyrus; STG, superior temporal gyrus; IFG, inferior frontal gyrus; SC-FC, structural-functional connectivity; *p < 0.01, **p < 0.001.

Significant inter-group differences were identified in brain regions across the frontal, temporal, parietal, insular, and limbic lobes involving most of the networks defined by Yeo et al. (42) (*p* < 0.01, 10,000 permutations, FDR corrected), and details were provided in **Table 4** and **Figure 3**. The subsequent *post-hoc* analyzes showed that (1) as compared to the HC group, the NSSI+ group exhibited significant increased SC-FC coupling in bilateral precuneus subregions, along with decreased SC-FC coupling in the right subgenual anterior cingulate cortex (sgACC), left superior temporal gyrus (STG), and right ventral insula (*p*s < 0.008, 10,000 permutations), (2) as compared to the HC group, the NSSI- group exhibited increased SC-FC coupling in the left dorsolateral superior frontal gyrus (SFG) (*p* = 0.007, 10,000 permutations), and (3) as compared to the NSSI- group, the NSSI+ group exhibited decreased SC-FC coupling in the left dorsolateral superior frontal gyrus (SFG), and increased coupling in the bilateral medial precuneus and the right opercular inferior frontal gyrus (IFG) (*p*s < 0.005, 10,000 permutations). Detailed information can be found in **Table 5** and **Figure 3**.

**Table 4:** Statistical comparisons of SDI values among the NSSI+, NSSI- and HC groups using one-way ANOVA.

Region	Side	Region label from 246 brain template	Modified cyto-architectonic descriptions	Yeo 7 network	MNI coordinate			*F_2,95_*	*p*
					x	y	z		
ACC	Right	CG_R_7_7	Subgenual area 32	DMN	5	41	6	9.7553	< 0.001^**^
Precuneus	Left	PCun_L_4_4	Area 31	DMN	-6	-55	34	9.3136	< 0.001^**^
Precuneus	Right	PCun_R_4_4	Area 31	DMN	6	-54	35	6.6888	< 0.001^**^
SFG	Left	SFG_L_7_4	Dorsolateral area 6	DAN	-18	-1	65	6.1933	< 0.001^**^
Precuneus	Right	PCun_R_4_1	Medial area 7	FPN	6	-65	51	6.1616	< 0.001^**^
STG	Left	STG_L_6_3	TE1.0 and TE1.2	SMN	-50	-11	1	6.1456	< 0.001^**^
Precuneus	Left	PCun_L_4_1	Medial area 7	FPN	-5	-63	51	5.8420	< 0.001^**^
IFG	Right	IFG_R_6_5	Opercular area 44	VAN	42	22	3	5.3084	< 0.001^**^
Insula	Right	INS_R_6_4	Ventral dysgranular and granular insula	VAN	39	-2	-9	4.8273	< 0.001^**^

SDI, Structural-decoupling index; NSSI+, suprathreshold nonsuicidal self-injury; NSSI-, subthreshold nonsuicidal self-injury; HC, healthy controls; ANOVA, analysis of variance; MNI, Montreal Neurological Institute; SFG, superior frontal gyrus; IFG, inferior frontal gyrus; STG, superior temporal gyrus; ACC, anterior cingulate cortex; DNA, dorsal attention network; VAN, ventral attention network; SMN, somatomotor network; FPN, frontoparietal network; DMN, default mode network. *p < 0.01, **p < 0.001.

**Table 5:** *Post-hoc* analyzes of the SDI values in the brain regions with significant inter-group differences.

Contrast	Region	Side	Region label from 246 brain template	*T*	*p*
NSSI+ *vs*. HC					
	ACC	Right	CG_R_7_7	4.6109	< 0.001^**^
	STG	Left	STG_L_6_3	3.2894	< 0.001^**^
	Insula	Right	INS_R_6_4	3.1410	0.002^*^
	Precuneus	Right	PCun_R_4_1	-2.7783	0.008^*^
	Precuneus	Left	PCun_L_4_1	-2.8370	0.007^*^
	Precuneus	Right	PCun_R_4_4	-3.2738	0.001^*^
	Precuneus	Left	PCun_L_4_4	-4.1877	< 0.001^**^
NSSI- *vs*. HC					
	SFG	Left	SFG_L_7_4	-2.8402	0.007^*^
NSSI+ *vs*. NSSI-					
	SFG	Left	SFG_L_7_4	3.1788	0.002^*^
	Precuneus	Left	PCun_L_4_1	-2.8432	0.005^*^
	Precuneus	Right	PCun_R_4_1	-3.1767	0.002^*^
	IFG	Right	IFG_R_6_5	-3.2855	0.001^*^

SDI, Structural-decoupling index; NSSI+, suprathreshold nonsuicidal self-injury; NSSI-, subthreshold nonsuicidal self-injury; HC, healthy controls; ACC, anterior cingulate gyrus; STG, superior temporal gyrus; SFG, superior frontal gyrus; IFG, inferior frontal gyrus. *p < 0.01, **p < 0.001.

### 3.3 Correlational analyzes

**Figure 4:**
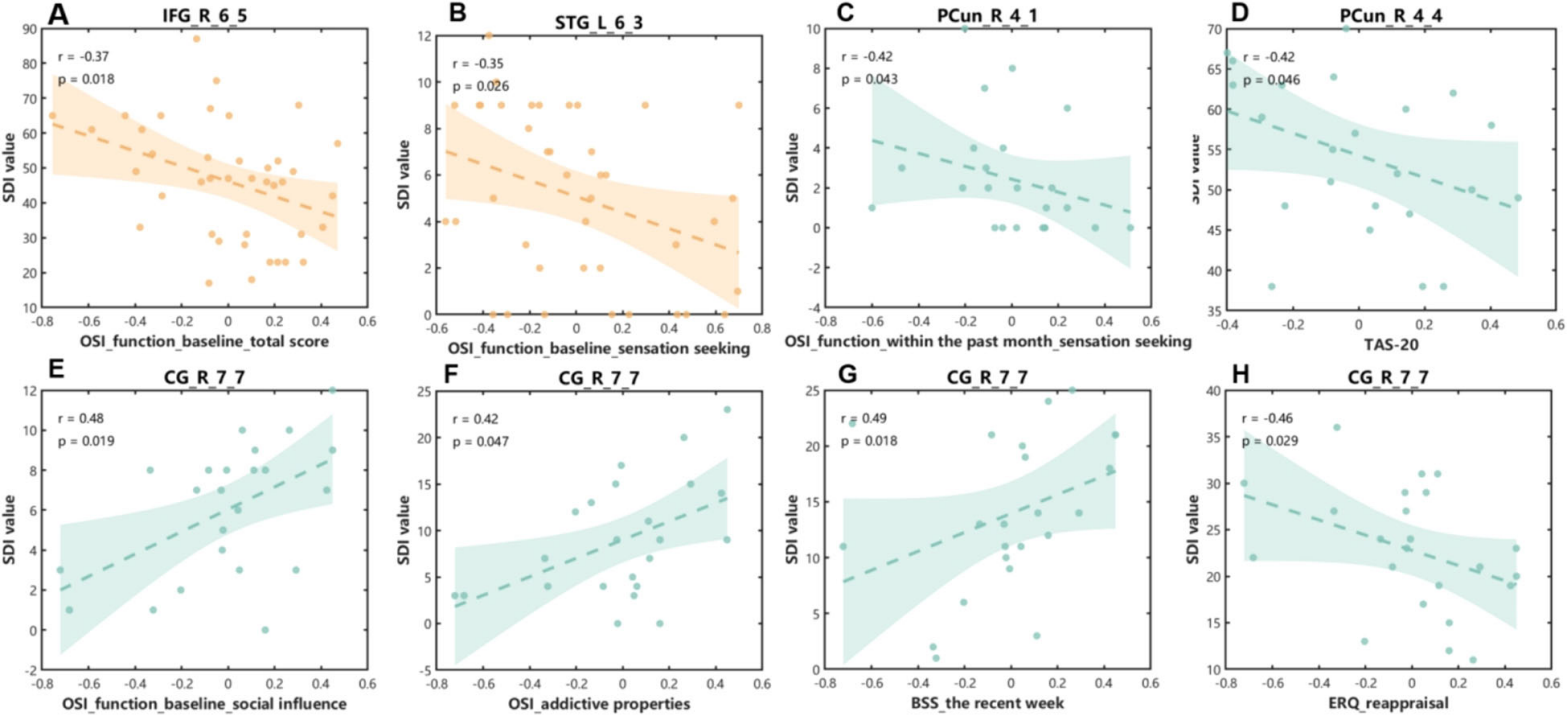
Correlations between the SDI values across brain regions with inter-group differences and the clinical variables. **(A, B)** In the NSSI+ group, the S DI value in the right opercular IFG (labeled as the IFG_R_6_5) was inversely correlated with the total score on the functions factors of NSSI at baseline (*r* = -0.37, *p* = 0.018), and the SDI value in the left STG (labeled as the STG_L_6_3) was inversely correlated with the score on the sensation-seeking factor of NSSI functions at baseline (*r* = -0.35, *p* = 0.026). **(C-H)** In the NSSI- group, the SDI value in the right medial precuneus (labeled as the PCun_R_4_1) was inversely correlated with the score on the sensation-seeking factor of NSSI functions within the past month (*r* = -0.42, *p* = 0.043), the SDI value in the right precuneus (labeled as the PCun_R_4_4) was inversely correlated with the TAS score (*r* = -0.42, *p* = 0.046), and the SDI value in the right sgACC (labeled as the CG_R_7_7) was positively correlated with the scores on the social influence factor of NSSI functions at baseline, the addictive features of NSSI, and the current suicidal ideation (*r* = 0.48, *p* = 0.019; *r* = 0.42, *p* = 0.047; *r* = 0.49, *p* = 0.018), respectively, and was negatively correlated with the score on the ERQ_reappraisal subscale (*r* = -0.46, *p* = 0.029). However, none of the correlations were significant after FDR multiple comparison corrections. SDI, structural-decoupling index; OSI, Ottawa Self-injury Inventory; TAS-20, Toronto Alexithymia Scale-20 items; BSS, Beck Scale for Suicide Ideation; ERQ, Emotion Regulation Questionnaire; IFG, inferior frontal gyrus; STG, superior temporal gyrus; sgACC, subgenual anterior cingulate cortex; FDR, false discovery ratio.

We explored the associations of SC-FC coupling, using the average SDI values in brain regions with inter-group differences, with the clinical variables in the two NSSI groups, respectively. In the NSSI+ group, the SDI value in the right opercular IFG was inversely correlated with the total score on the functions factors of NSSI at baseline (*r* = -0.37, *p* = 0.018), and the SDI value in the left STG was inversely correlated with the score on the sensation-seeking factor of NSSI functions at baseline (*r* = -0.35, *p* = 0.026). In the NSSI- group, the SDI value in the right medial precuneus was inversely correlated with the score on the sensation-seeking factor of NSSI functions within the past month (*r* = -0.42, *p* = 0.043), the SDI value in the right precuneus (area 31) was inversely correlated with the TAS score (*r* = -0.42, *p* = 0.046), and the SDI value in the right sgACC was positively correlated with the scores on the social influence factor of NSSI functions at baseline, the addictive features of NSSI, and the current suicidal ideation (*r* = 0.48, *p* = 0.019; *r* = 0.42, *p* = 0.047; *r* = 0.49, *p* = 0.018), respectively, and was negatively correlated with the score on the ERQ_reappraisal subscale (*r* = -0.46, *p* = 0.029). However, none of the correlations were significant after FDR multiple comparison corrections. Detailed information was provided in **Figure 4**.

## 4 Discussion

This study represents a significant advancement in understanding the neural mechanisms underlying NSSI among depressive female adolescents. By classifying self-injurious participants into NSSI+ (meeting DSM-5 criteria) and NSSI- (without meeting DSM-5 criteria) groups, we aimed to elucidate distinct neural correlates associated with these subtypes. Our analysis focused on SC-FC coupling, providing novel insights into the neurobiological differences between these groups.

The primary finding of our study is the identification of specific brain regions where SC-FC coupling differs significantly between NSSI+ and NSSI- groups. These regions include the left dorsolateral SFG, right opercular IFG, and bilateral medial precuneus. Based on the Yeo 7-network framework, these areas are predominantly implicated in the brain’s dorsal attention network (DAN), ventral attention network (VAN), and frontoparietal network (FPN), all of which are typical task-positive networks and play critical roles in cognitive control, attentional processes, and goal-directed behaviors (43, 44).

The left dorsolateral SFG, overlapping with the dorsolateral prefrontal cortex (DLPFC), showed a more pronounced state of SC-FC decoupling in the NSSI+ group compared to the NSSI- group. The DLPFC is central to “cold” executive functions—higher-order cognitive operations that are relatively independent of emotional or reward-based input, such as working memory, goal-oriented planning, attentional control, and response inhibition (45–47). In contrast, “hot” executive functions involve affective or reward-related processing. Prior work has linked NSSI in depression to impairments in cognitive control, including reduced goal-directed behavior (48) and diminished cognitive flexibility (49), along with altered DLPFC activity during cognitively demanding tasks (50–52). The observed decoupling in the left dorsolateral SFG may therefore reflect gradations in cognitive-attentional and executive processing underlying different severity levels of NSSI in depressed adolescent females.

Similarly, the right opercular IFG, part of the VAN, exhibited increased SC-FC coupling in the NSSI+ group relative to the NSSI- group. The VAN is crucial for bottom-up attentional processes, such as attentional shifts (43). The IFG has reliable connections with both the supplementary motor area (SMA), supporting its crucial involvement in flexible cognitive-motor inhibition, such as the inhibition of premature or no longer appropriate motor responses (53, 54). Previous studies have reported reduced right IFG volume and abnormal error-related activation during Go/No Go task in individuals engaging in self-injurious behaviors (55, 56). Our results extend these observations by highlighting differences in SC-FC coupling properties of the right opercular IFG, which may indicate variations in cognitive motor inhibition functions among depressive female adolescents with different levels of self-injury severity. Furthermore, a negative correlation emerged between the extent of decoupling in this region and the baseline total score of NSSI functions within the NSSI+ group. We postulate that the functions or motivations underlying self-injurious behavior could cognitively intensify such behavior, resulting in subsequent difficulties in inhibiting self-harm. This process may be intricately linked to an aberrant over-coupling of structure and function in the right opercular IFG.

The precuneus was initially considered a brain region primarily associated with visual processing. Recent imaging studies highlight its more crucial functions in complex cognitive processes, including risk/reward assessment, working memory updating, and self-referential thinking, due to its extensive connections with large-scale brain networks such as the default mode network (DMN) and FPN (57). Our findings showed increased SC-FC coupling of the bilateral medial part of precuneus, which is intricately involved in the FPN (58, 59), in the NSSI+ group compared to the NSSI- group. Moreover, our study found a negative link between the decoupling degree of the right medial precuneus and the sensation-seeking factor of NSSI functions in the recent month within the NSSI- group. Sensation-seeking, a core trait in addictive disorders, has been linked to NSSI, which some view as a behavioral addiction (60, 61). Similarly, patients with substance addiction have shown to exhibit medial precuneus and FPN abnormalities (62, 63). Thus, it is plausible that the abnormal precuneus coupling might underlie vulnerability to sensation-seeking-induced self-injury behavior in less severe NSSI individuals. Further research is needed to clarify if this extends beyond pain perception. Interestingly, Baum et al. (64) reported that age-related increases in coupling were particularly prominent within brain regions of the DMN and FPN, including the precuneus. They emphasized that delayed development of coupling in these brain networks might provide a critical window for neural remodeling during childhood and adolescence. It is plausible that the excessive SC-FC coupling of the precuneus observed in NSSI+ adolescents in our study may reflect impaired neural plasticity, which could contribute to the escalated severity of self-injury behavior.

It has been summarized that neural structural and functional alterations in NSSI mainly involve the fronto-limbic-striatal system (65). Xu et al. (32) found increased SC-FC coupling in the right insula and left thalamus among depressed adolescents without NSSI behaviors compared to those with NSSI and HCs. In our study, we demonstrated that the depressive female adolescents with NSSI+ behavior showed widespread coupling abnormalities spanning the somatomotor network, FPN, DMN, and VAN versus the HC group, while those with NSSI- behavior only in the left dorsolateral SFG. These findings underscore subthreshold NSSI as a potential critical period for intervention, during which neurobiological alterations may still be reversible. Notably, we observed a dissociation in the left dorsolateral SFG: while the NSSI- group differed from HC in this region, the NSSI+ group did not. This paradoxical finding suggests a potential compensatory mechanism in which initial coupling disruptions (seen in NSSI- individuals) may normalize or shift to other regions as NSSI behaviors escalate.

Interestingly, correlational analyzes revealed several significant associations between decoupling indices and clinical features, especially in the right sgACC among the NSSI- participants, a key region which has recently been found to be able to enhance the precision and therapeutic efficacy of DLPFC targeting for depression treatment (66–68). Given the common comorbidity of depressive symptoms in NSSI adolescents and the empirical evidence supporting that the alleviation of depressive symptoms contributes to NSSI behavior improvement (69, 70), our findings highlight a promising approach, using dual-region modulation of superficial DLPFC and deeper sgACC via repetitive transcranial magnetic stimulation (rTMS), for early intervention of NSSI.

## 5 Limitations

Certain limitations must be acknowledged in the present study. First, only female participants were included, and whether similar brain alterations exist in male self-injury populations remains unknown. Second, the majority of NSSI participants in this study had a background of depression. It is difficult to exclude the influence of affective disorders due to the absence of a non-NSSI depressive group. Third, while psychiatrists grouped participants according to the suggested diagnostic criteria for NSSI in DSM-5, there is currently no Structured Clinical Interview for DSM-5 specifically targeting NSSI diagnosis. Therefore, the subjective influence of psychiatrists cannot be entirely ruled out during group classification. Lastly, this study cannot determine whether the abnormal SC-FC coupling represents a state feature or a trait feature of different NSSI subtypes. Future research needs to delve deeper into the association between the brain SC-FC coupling characteristics and the dynamic changes in NSSI behaviors from a longitudinal perspective.

## 6 Conclusions and future directions

The present study demonstrates that SC-FC coupling analysis offers a sensitive, integrative framework for distinguishing between clinically relevant subtypes of NSSI in female adolescents with depression. By comparing individuals whose NSSI meets the DSM-5 diagnostic threshold with those exhibiting subthreshold symptoms, we identified subtype-specific alterations in SC-FC coupling within key cortical regions—including the left dorsolateral SFG, right opercular IFG, and bilateral medial precuneus. These regions are predominantly embedded in the task-positive networks, which collectively support executive control, attentional allocation, and goal-directed behavior. Importantly, the NSSI+ group exhibited more extensive and severe coupling abnormalities, whereas the NSSI- stage may represent a critical window for early intervention during which neural dynamics may still be modifiable. The right sgACC, which demonstrated significant correlations with several clinical features in the NSSI− group, emerges as a particularly promising neurobiological target for early intervention strategies.

To build upon the present findings, future research should adopt longitudinal designs to clarify whether SC-FC coupling alterations reflect state or trait characteristics of NSSI, and expand investigations to include males and non-depressed NSSI populations to improve generalizability. The development of standardized diagnostic tools for NSSI would enhance classification reliability, while studies linking baseline coupling profiles to treatment outcomes—particularly through targeted neuromodulation aimed at normalizing aberrant coupling patterns—could advance personalized intervention strategies. Further integration of multimodal data and translational applications may ultimately support the use of SC-FC coupling as a biomarker for risk stratification and treatment monitoring in youth with NSSI.

## Data Availability

The raw data supporting the conclusions of this article will be made available by the authors, without undue reservation.
